# Israeli Spotted Fever, Tunisia

**DOI:** 10.3201/eid1707.101648

**Published:** 2011-07

**Authors:** Abir Znazen, Boussayma Hammami, Dorra Lahiani, Mounir Ben Jemaa, Adnene Hammami

**Affiliations:** Author affiliations: Habib Bourguiba University Hospital, Sfax, Tunisia (A. Znazen, A. Hammami) and Hédi Chaker University Hospital, Sfax (D. Lahiani, M. Ben Jemaa, B. Hammami)

**Keywords:** bacteria, spotted fever group, rickettsiae, Israeli spotted fever, Tunisia, letter

**To the Editor:** Mediterranean spotted fever (MSF) caused by *Rickettsia conorii* was the first rickettsiosis described in Tunisia. *R. conorii* was thought to be the only species existing in this country. However, authors reported other rickettsioses either from spotted fever group or typhus group (*1**,**2*). In Sfax, a town in southern Tunisia, physicians noted patients with severe forms of MSF and suspected the presence of other species or a virulent *R. conorii* strain. We report 2 cases of Israeli spotted fever (ISF) from Sfax that were confirmed by detection of rickettsial DNA in skin biopsy specimens.

In September 2009, two previously healthy men, 45 and 46 years of age (patients 1 and 2, respectively), were hospitalized in the infectious disease department of Hedi Chaker University Hospital (Sfax, Tunisia). They came from suburban areas 30 km apart. The men were admitted with histories of fever of a few days’ duration and cutaneous maculopapular rash. Patient 1 had fever of 38°C, chills, headache, and arthromyalgia without hemodynamic abnormalities. Patient 2 was admitted with fever of 41°C, conjunctivitis, and cardiovascular collapse; he was treated in an intensive care unit for 1 day. No inoculation eschar was found on either patient. Biological findings for the 2 patients showed a leukocyte count within normal ranges, anemia, thrombopenia, high levels of C-reactive protein, and elevated liver enzymes. Both patients had contact with dogs, but neither patient reported a tick bite. The patient with more severe illness worked in the livestock importation industry; his illness developed 5 days after his return from a 2-week trip to Libya. The patients received 200 mg doxycycline per day for 10 days and improved rapidly.

Skin biopsy specimens from the rash and whole blood samples were obtained from the 2 patients. PCRs targeting outer membrane protein (*omp*) *A* and *B* genes were done by using previously described primers (*3*). A negative control (sterile water and DNA from a sterile biopsy specimen) and a positive control (*R. montanensis* DNA) were included in each test. Amplicon sequencing confirmed *R. conorii* ISF strain DNA in the 2 skin samples and in the blood sample of patient 1. For both patients, the sequence homology to *R. conorii* ISF strain DNA was 99% for *ompB* gene (833 pb) and 100% for *ompA* (596 pb) (GenBank accession nos. AF123712.1 and AY197564.1, respectively). Serologic testing performed by a microimmunofluorescence assay yielded negative results for the first blood samples. A second blood sample was tested only for patient 2 and showed immunoglobulin M titers of 64 and immunoglobulin G titers of 128.

We demonstrated human infection caused by *R. conorii* ISF strain in Tunisia. This strain has been recently suggested for classification with 3 others as a subspecies within the species *R. conorii* on the basis of multilocus sequence typing (*4*). ISF was first described in Israel where it is endemic (*5*). The disease appears to be more widely spread in the Mediterranean countries than first believed because cases from Italy and Portugal have been reported (*6**,**7*). Recently, a patient from Switzerland with confirmed ISF was suspected to be infected in Libya (*8*). One patient in our study may also have been infected during his stay in Libya. Thus, geographic distribution of ISF seems to be extended to all Mediterranean countries and not limited to Israel, Italy, and Portugal. Its distribution areas probably overlap with those of MSF because the 2 infections share the same vector, the dog tick (*Rhipicephalus sanguineus*) (*9*). Although a history of tick bite could not be documented from the recorded anamnesis data, contact with dogs was noted in our cases. Furthermore, the 2 cases were diagnosed during the same month (September), corresponding to seasonal fluctuations generally observed for MSF in our region. Although 1 of our patients reported recent travel, the second patient affirmed he had not left his locality; thus, endemicity of ISF in our region in Tunisia is possible.

De Sousa et al. reported the differences between patients infected with *R. conorii* Malish and ISF strains (*10*). The characteristic eschar at the site of the tick bite was markedly less noted in ISF. The absence of this eschar has been also described in other studies (*9**,**10*). In our report, patients were treated with a delay of 10 and 6 days and neither patient died, but 1 patient did experience severe illness. Our observations suggest that the supposed ability of the ISF *Rickettsia* sp. to cause more severe illness is not ascribed to late diagnosis but may be due to more virulent strains, as suspected by De Sousa (*10*).

Finally, PCR applied to whole blood and tissue samples was more effective in diagnosing these cases earlier than serology because antibodies appear to have slow kinetics. Physicians should be alert to the possibility of ISF in febrile patients in our region, especially because fatal outcomes of this infection have been reported (*8*).
